# P. R. Cortelyou, M. D., Marietta, GA.

**Published:** 1890-01

**Authors:** 


### Clinical Department.

P. R. CORTELYOU, M. D., MARIETTA, GA.

### CHOREA.

The patient consulted me April 2d, 1889, and his mother gave the history, e. a., aged 16 years, had suffered from an attack of acute rheumatism some six months before this time. After convalescence from this attack, the nervous symptoms began to appear, and had gradually grown more severe. He had been under treatment for about two months but had not improved.

The patient was very enemic and had no control over his muscles, but his arms, face and legs were in continual action. It was with difficulty that he could walk or feed himself. He was placed on the following mixture:

| | | |
|---|---|---|
| ℞ | Tinct. ferri chloride, | ℥p |
| | Potass bromide, | ʒiip |
| | Liq. potass arsenit, | ʒii |
| | Aqual qs. ad. | ℥iv |

Sig. One teaspoonful in water to be taken after each meal. On this line of treatment, his improvement was very rapid, and in four weeks he was entirely relieved, having gained in flesh, having good healthy color, and free from muscular contortions. He has remained in good health since that time.

### CARBUNCLE.

Called to see patient, August 9th, 1889. M. J., colored, married, about 55 years of age. Found her suffering with large carbuncle under right scapula. The tumors were very much indurated, covering space of four and one-half inches in diameter. Had been about eight days since commencment of the carbuncle. Patient suffering good deal, unable to sleep, and feeling very weak. I injected the center of the tumor with following:

| | | |
|---|---|---|
| ℞ | Acid carbolic | xx |
| | Glycerine, g. | ʒj |
| | Aqua, | ʒvii |

Using one drachm of the solution, and injecting in three places one-third of the quantity in each place. The injection caused considerable pain, but in a few minutes, the patient said she was easier and freer from pain than she had been for several days. The tumor was then thickly covered with following:

| | | |
|---|---|---|
| ℞ | Acid carbolic | gtts xx |
| | Acid boracic | ʒii |
| | Fl. ext. ergot | ʒii |
| | Oxid. zinc | ʒii |
| | Vaseline | ℥iii |
| M. | | |

Also gave pills of sulphide of calcium gr. one-fifth three times a day. August 10. The tumor was again injected with carbolic solution. This treatmen was continued for four or five days, when the center had softened and discharged freely. From this on she made rapid recovery.

From the result in this case I was much pleased with the action of the carbolic solution hypodermically; not only from the decided relief from pain given by the injection, but also as I believe from the antiseptic effect of the solution, limiting the destruction of tissue, by destroying the anthrax microbe. The patient recovering in ten days days treatment from the effect of a large carbuncle, which by the old method of crucial incisions through the tumor would have caused the loss of much blood, in an already debilitated subject, and would have delayed very much the recovery.

INTERNAL HEMORRHOIDS.

Mrs., married, about 35 years of age. The patient had suffered for many years from hemorrhoids, with frequent bleeding and protrusion of the tumors. In August 1888, the bleed-being very free and frequent, she had tumors injected with carbolic acid solution. This checked the hemorrhages, but did not destroy the hemorrhages. Examination showed four or five tumors about the size of an English walnut protruding from the anus—with much relaxation of the surrounding mucous membrane. She was not able to stand or walk much with the tumors protruding. Removal was advised, and patient consenting, Dr. Willis Westmoreland, of Atlanta, attended by Dr. Johnson and myself, operated August 5th, 1889, using the clamp and cautery. The operation was quickly and thoroughly made, and the patient recovered without any delay or complications, obtaining complete relief. Much has been written in regard to the merits of the various operations for internal hemorrhoids, but this operation as performed by Dr. Westmoreland in efficiency, quickness and safety seems to be all that can be desired.

---

### FACIAL NEURALGIA.

Dr. J. McFadden Gaston referred to two cases of facial neuralgia.

One was presented in the person of a man about thirty years old, with a previous history of some hemicrania and enlargement of the cervical gland, with an indurated protuberance on the left side beneath the ear, presenting internal obliquity in the left eye, and diviation of the tongue to the left when protruded. There had been occasionally enlargment of the tonsils, but no sign of permanent induration.

He had taken quinine in liberal doses for several days in succession repeatedly without notable effect. Supposing that some deposit near the origin, or in the course of the nerve might be pressing upon it and causing the pain and inflammatory developments, an alterative course was adopted, giving calomel and Dovers powders internally and applying to the protuberance a combination of mercurial and belladonna oint. This was continued until ptyalism ensued, when a solution of chlorate of potash was used internally and as a gargle, with the local application of a liniment of spts. turp. and olive oil, each one ounce with camphor one drachm. After the subsidence of the salivation and tonsilitis, he was put upon the following: Iodide potash one-half ounce, succus alterans 4 ounces, one teaspoonful with half a glass of water three times a day. Externally: Iodine grs. x, camphor one drachm, collodian one ounce, apply daily with camel's hair pencil over the tumors.

This treatment was kept up for several weeks and in the meantime, the patient left the Infirmary and went to his home in the country near one of our neighboring towns, coming here for examination every week or ten days. There was little improvement and he concluded, two weeks ago, to suspend this treatment for the use of the following prescription:

℞ Antipyrin - - - ʒ iii
Ext. canab. Ind. - -
Ext. Aconite aa grs vss
Cafein - - - - ʒ ss
Hyoscine hydrob. - - gr i
Mix in 30 capsules
Take one three times a day.

The patient reported on yesterday that he had finished the medicine three days previously, and upon making an examination, notable aggravation of his constitutional condition was revealed. The pulse was frequent and feeble, with rise of temperature, while the deviation of the tongue from the medium line was increased, with some impairment of speech, diminution of the pain. He was prescribed 20 drops of tinct. digitalis, three times a day, with the hope of restoring tone to the heart, but realizing that the trouble was not from the medication, there is cause for serious apprehension as to the result of the case.

Note.—This patient, reported two days afterward, by Dr. Gaston, with a pulse of 130 per minute. Temp. 100. and increase of lingual paralysis, and the 20 drops of tinct. digitalis ordered every six hours. But a later examination showed improvement, with pulse of 100 and temperature normal, when he was ordered quinine and Fowler's arsenical solution.

The other case of facial neuralgia, was in a lady some fifty years old, who presented herself, requesting a division of the facial nerve with a hope to be relieved of her sufferings, which had continued for many years, and for which a deep incision had been made to the left of the nose, at a former period without any benefit. Finding that she had become addicted to use of morphine, hypodermically, in quantities of 15 grains a day, she was first put upon regulated doses of 2 grains every six hours, and eventually to every twelve hours, being morning and night. This reduction was attended with marked disturbances of the stomach and bowels, which necessitated stopping a prescription given at the outset as follows:

℞ Fowler's solution of arsenite of potash f ʒ ii
Succus alterans - - - f ℥ iv
Mix and take one teaspoonful three times a day.

In the meantime, to meet the indications of hepatic derangment she took the following combination: Blue mass, grs. xvii, comp. extract colocguth, grs. xii, podophylin, grs. iii, divided in six pills, one night and morning. This was attended with a good result. The persistent wretching was relieved by a prescription of lime water 3 ounces, camphor water 2 ounces, peppermint water 1 ounce, in tablespoon doses every hour or two. She then retured to the formula of antipyrin, etc., with benefit and is consequently free from trouble at present, and the quantity of morphine is being gradually diminshed.

